# Draft Genome Sequence of *Streptococcus gordonii* Type Strain CCUG 33482^T^

**DOI:** 10.1128/genomeA.00175-16

**Published:** 2016-03-24

**Authors:** Francisco Salvà-Serra, Hedvig E. Jakobsson, Kaisa Thorell, Lucia Gonzales-Siles, Erika T. Hallbäck, Daniel Jaén-Luchoro, Fredrik Boulund, Per Sikora, Roger Karlsson, Liselott Svensson, Antoni Bennasar, Lars Engstrand, Erik Kristiansson, Edward R. B. Moore

**Affiliations:** a Culture Collection University of Gothenburg (CCUG), Sahlgrenska Academy, University of Gothenburg, Gothenburg, Sweden; b Department of Infectious Diseases, Sahlgrenska Academy, University of Gothenburg, Gothenburg, Sweden; c Microbiology, Department of Biology, University of the Balearic Islands, Palma de Mallorca, Spain; d Department of Microbiology, Tumor and Cell Biology, Karolinska Institute, Stockholm, Sweden; e Department of Mathematical Sciences, Chalmers University of Technology, Gothenburg, Sweden; f Integrative Clinical Genomics, Clinical Diagnostics, SciLifeLab, Stockholm, Sweden; g Laboratory Medicine, Sahlgrenska University Hospital, Gothenburg, Sweden; h Nanoxis Consulting AB, Gothenburg, Sweden

## Abstract

*Streptococcus gordonii* type strain CCUG 33482^T^ is a member of the *Streptococcus mitis* group, isolated from a case of subacute bacterial endocarditis. Here, we report the draft genome sequence of *S. gordonii* CCUG 33482^T^, composed of 41 contigs of a total size of 2.15 Mb with 2,061 annotated coding sequences.

## GENOME ANNOUNCEMENT

*Streptococcus gordonii* is a commensal bacterium commonly found in human oral cavities and pharynges (1) and has been implicated in the formation of dental biofilms (2). *S. gordonii* also has been isolated from samples of bacterial endocarditis (3). *S. gordonii* was described as a novel species in 1989 after analyses of viridans streptococci (4). In those studies, *Streptococcus sanguis* type I-II, strain SK3^T^ (=CCUG 33482^T^ ←NCTC 7865^T^ ←ATCC 10558^T^), isolated from a patient with subacute bacterial endocarditis (5), was reclassified as a distinct species, *S. gordonii*, with strain SK3^T^ designated the type strain. *S. gordonii* is one of 14 species within the *Streptococcus mitis* group (6).

*S. gordonii* CCUG 33482^T^ was cultivated with 5% CO_2_, at 37°C, on Columbia agar base plus 5% horse blood. A fresh, pure-culture biomass was lysed in low-TE buffer (1 mM Tris, 0.1 mM EDTA, pH 8.0) supplemented with Triton 1.2% (wt/vol), lysozyme (20 mg/ml), and proteinase K (1 mg/ml), at 56°C overnight. Genomic DNA was isolated, using a MagNA pure compact nucleic acid isolation kit version I (Roche Diagnostics, Mannheim, Germany). DNA was sequenced with an Illumina MiSeq instrument (SciLifeLab, Stockholm, Sweden), generating 633,858 paired-end reads of 300 bp, yielding a total of 190.2 Mb. Sequence reads were trimmed and assembled *de novo* with CLC Genomic Workbench version 8 (CLC bio, Aarhus, Denmark) and default settings. Assembly quality was assessed using Quast version 3.1 (7). Trimming of the sequence reads left 632,358 paired-end reads (80.2% of initial sequence data) with an average of 241 bp, producing an assembly length of 2,166,763 bp, consisting of 41 contigs; the longest contig measures 632,335 bp. The *N*_50_ is 233,538 bp, and the average genome coverage of the assembly is 71×. The G+C content of the genome sequence is 40.5%, which is consistent with the 41% reported when the species was described (4).

The genome sequence was annotated using the NCBI Prokaryotic Genome Annotation Pipeline (PGAP) (8). Annotation identified 2,061 coding sequences, 46 tRNAs, and 3 repeat regions. Comparative analyses of the sequence, by average nucleotide identity based on BLAST (ANIb) (9), using JSpecies version 1.2.1 (10), with the genome sequences of the type strains of species of the *S. mitis* group ranged from 73.1% to 81.8%. The analysis of the genome sequence for the presence of CRISPR-Cas systems (11) with CRISPRFinder (12) confirmed the presence of 4 CRISPR regions. Analyses against the Comprehensive Antibiotic Resistance Database (CARD) (13) and against the virulence factor database (VFDB) (14) identified 8 putative antibiotic resistance genes and 72 putative virulence factors. At the beginning of this project, the genome sequence of the type strains of all species included within the *S. mitis* group were available, except for that of *S. gordonii*. The genome sequence of *S. gordonii* CCUG 33482^T^ represents essential data for the characterization of metabolic features of *Streptococcus* and phylogenomic studies of the *S. mitis* group.

### Nucleotide sequence accession numbers.

This whole-genome shotgun project has been deposited at DDBJ/ENA/GenBank under the accession number LQWV00000000. The version described in this paper is the first version, LQWV01000000.
